# Anesthetic Management of Awake Craniotomy Versus Traditional Craniotomy at a Single Academic Center: A Retrospective Review

**DOI:** 10.7759/cureus.81344

**Published:** 2025-03-28

**Authors:** Lakshmi N Kurnutala, Vikas Chauhan, Richard S Smith, Michelle Tucci

**Affiliations:** 1 Anesthesiology, University of Mississippi Medical Center, Jackson, USA

**Keywords:** awake craniotomy, length of stay, morphine milligram equivalents, neuromonitoring, traditional craniotomy

## Abstract

Awake craniotomies (AC) are an increasingly popular surgical technique used in medical institutions worldwide. This rise in adoption is largely due to improved patient outcomes and satisfaction. This procedure allows for real-time monitoring of neurological functions, which helps surgeons preserve critical cognitive, language, and motor abilities. As a result, patients tend to retain neurological functions closer to their baseline levels, highlighting the technique's advantages in modern neurosurgery. Neuromonitoring and brain mapping allow surgeons to perform resections more precisely in delicate anatomical areas. We developed and implemented a protocol for awake craniotomy procedures at the University of Mississippi Medical Center a few years ago. The purpose of this study was to assess and analyze the data between anesthetic management of awake craniotomy and traditional craniotomy (TC). We compared patient characteristics, anesthetic management, surgical duration, complications, opioid requirements, and length of hospital stay (LOS). Our results indicated a statistically significant reduction in the opioid requirement for patients in the awake craniotomy group with no difference in the overall length of stay.

## Introduction

Awake craniotomy (AC) procedures have been performed for hundreds of years, with the first documented case dating back thousands of years [1,2]. Archaeological evidence suggests that humans performed successful brain surgeries while patients were conscious long before modern anesthesia existed. This is demonstrated by ancient Peruvian skulls, where over half of the 214 examined specimens showed signs of complete healing after trepanation procedures. Some researchers hypothesize that local anesthesia may have been achieved using cocaine from coca leaves. During the 19th century, the understanding of cerebral localization advanced [2]. The contemporary approach to awake brain surgery was pioneered by Penfield and Pasquet in their groundbreaking research from the mid-20th century. Since then, awake craniotomies have become an important surgical option, especially for treating brain tumors.

With the advancement of anesthetic techniques, neuromonitoring, and improvement in brain mapping modalities, surgeons can now remove tumors with greater precision to minimize neuronal injury [3,4]. Several significant developments have marked the evolution of AC techniques. These include enhanced neurophysiological monitoring capabilities [5], advanced anesthetic protocols allowing for intraoperative rapid awakening and cognitive assessment [6], and a better understanding of brain plasticity and functional mapping [7], the remarkable enhancements in neurophysiological monitoring capabilities [5], which now allow for real-time assessment of brain function during surgery.

Proper patient selection is essential to reduce perioperative morbidity and mortality [8]. The presence of a difficult airway, obesity, and obstructive sleep apnea (OSA) can make it uniquely challenging. During the phase where patients are awake during the ongoing surgery, other factors can become critical for the successful conduct of the procedure. These are psychological preparation and emotional stability [9]. The patient needs to cooperate to facilitate the assessment of the desired neurological function [10]. The anatomical considerations of the lesion, such as location, can make patient positioning more challenging and also affect the surgical approach and risks involved [11]. The presence of drapes over the patient, lack of mobility, and the overall environment of the operation room can make the experience acutely challenging for patients with severe anxiety or claustrophobia [12].

With the increased utilization of the AC technique, the role of the anesthesiologist has become paramount in ensuring the success of these procedures. The anesthesiologist's role has evolved to provide titrable analgesia and sedation with varying depths throughout the surgery and also ensure patient safety during ongoing surgery, with particular attention to respiratory, hemodynamic, and neurological function [13]. Continuous intraoperative communication between the surgeon, anesthesiologist, neurophysiology team, and neuropsychologist is an integral part that helps to improve the overall safety and success of awake craniotomies [14]. Other advances include the development of specialized drug protocols [15], the implementation of targeted sedation scales [16], enhanced monitoring techniques [17], and standardized communication protocols [18].

Since developing our protocol in 2020 for awake craniotomies at the University of Mississippi Medical Center, numerous procedures have been performed over the last few years. This study was designed to compare, assess, and analyze the data between the anesthetic management of awake craniotomy and traditional craniotomy (TC).

## Materials and methods

This retrospective chart review study was completed at a single institution and included craniotomies done between December 2020 and August 2022. The Institutional Review Board (IRB) of the University of Mississippi Medical Center (UMMC-IRB-2022-326) approved the study protocol. During this study duration, data was collected for patients getting awake craniotomies and traditional craniotomies, which matched the inclusion criteria.

This study aimed to assess and analyze the data between the anesthetic management of awake craniotomy and traditional craniotomy. We compared patient characteristics, anesthetic management, surgical duration, opioid requirements, and length of hospital stay (LOS).

The institutional protocol followed for the anesthesia used for awake craniotomy is presented in the Appendices. It can be described briefly as scalp block at the start with ropivacaine and lidocaine and the use of propofol, remifentanil, and dexmedetomidine infusions titrated to the patient's anesthetic requirement.

For traditional craniotomies, the anesthesia constituted general anesthesia with endotracheal intubation, with the use of standard anesthesia medications in appropriate doses with propofol, rocuronium, and fentanyl, along with sevoflurane. Reversal of muscle relaxation was done with sugammadex. All patients across two study groups received intraprocedure standard American Society of Anesthesiology (ASA) monitoring along with an invasive arterial line for blood pressure management.

Table 1 shows the inclusion and exclusion criteria.

**Table 1:** Patient selection: inclusion and exclusion criteria ASA: American Society of Anesthesiology

Inclusion criteria		Exclusion criteria	
1	Patients over 18 years of age	1	Age under 18 years
2	Scheduled for either awake craniotomy or traditional craniotomy	2	Pregnant women
3	Primary brain tumor or epilepsy surgery candidates	3	Emergency surgeries
4	ASA physical status I-III	4	Severe cognitive impairment
		5	History of adverse reactions to local anesthetics

Data was extracted from electronic medical records using a standardized data collection form. Variables collected included demographic information (age, gender, and body mass index (BMI)), ASA physical status, primary diagnosis, surgical approach and duration, anesthetic management details, perioperative complications, opioid consumption (converted to morphine milligram equivalent (MME)), length of hospital stay, and postoperative neurological outcomes.

After a comprehensive data review of the awake and traditional craniotomies meeting our inclusion criteria, 27 patients were included in the awake craniotomy (AC) group and 26 in the traditional craniotomy (TC) group. One neurosurgeon performed all the awake craniotomies, and the same surgeon performed most of the traditional craniotomies. However, to increase the number of controls, some traditional craniotomies performed by a second neurosurgeon at the same institution were included.

After data collection was complete and entered into REDCaps®, statistical analysis was performed using SPSS version 26.0 (IBM Corp., Armonk, NY). Continuous variables were expressed as mean ± standard error of the mean (SEM). Categorical variables were expressed as frequencies and percentages. Comparisons between the groups were performed using independent samples t-test for continuous variables, Chi-square or Fisher's exact test for categorical variables, and Mann-Whitney U test for non-normally distributed data. A p-value < 0.05 was considered statistically significant.

## Results

Our analysis revealed comparable baseline characteristics between the two groups (Table 2). There was no significant difference (AC: 49.89 ± 5.41 years versus TC: 51.85 ± 6.35 years, p = 0.633). When comparing the sex of both groups, we found no difference (AC: 16:11 versus TC: 11:15 (male:female), p = 0.165). Similarly, BMI (AC: 28.97 ± 2.64 kg/m² versus TC: 30.17 ± 3.14 kg/m², p = 0.551) and ASA classifications (AC: 2.89 ± 0.17 versus TC: 3.27 ± 0.22, p = 0.005) were statistically similar between both the groups compared.

**Table 2:** Comparison between the AC and TC groups AC: awake craniotomy, TC: traditional craniotomy, SEM: standard error of the mean, BMI: body mass index, ASA: American Society of Anesthesiology, MME: morphine milligram equivalent, mg: milligrams *: p-value ≤ 0.05 (statistically significant)

	AC (mean ± SEM)	TC (mean ± SEM)	p-value
Age (years)	49.89 ± 5.41	51.85 ± 6.35	0.633
BMI (kg/m^2^)	28.97 ± 2.64	30.17 ± 3.14	0.551
ASA status	2.89 ± 0.17	3.27 ± 0.22	0.005*
Intraoperative MME (mg)	365.13 ± 94.91	683.95 ± 211.99	0.006*
Duration of surgery (minutes)	219.07 ± 21.42	169.0 ± 27.29	0.004*
In-hospital MME/day (mg)	9.17 ± 5.72	25.77 ± 15.55	0.042*
Length of stay (days)	7.07 ± 2.40	5.15 ± 1.24	0.153

The operating time was significantly longer in the AC group (AC: 219.07 ± 21.42 versus TC: 169.0 ± 27.29 minutes, p = 0.004) (Table 2). We found statistically significant differences in opioid consumption. During the intraoperative period, the AC group had an opioid requirement of 365.13 ± 94.91 MME, and the TC group had 683.95 ± 211.99 MME, with a p-value of 0.006 on comparing these two (Table 2). The daily in-hospital opioid requirements were also less in the AC group (9.17 ± 5.72 MME/day) than the TC group (25.77 ± 15.55 MME/day) (p = 0.042) (Table 2). The overall length of stay, however, showed no significant difference (AC: 7.07 ± 2.40 days versus TC: 5.15 ± 1.24 days, p = 0.153) (Table 2). As shown in Table 2, there was a statistically significant difference in the ASA status between these two groups. There were two outliers in the AC group who experienced a prolonged length of stay. One case was of postoperative stroke, and the other one was of worsening motor deficits requiring rehabilitation.

We encountered a few complications specific to the AC group. These were loss of vascular access (IV and arterial lines, one patient), airway obstruction requiring supraglottic device insertion (laryngeal mask airway (LMA) was inserted, one patient), procedure intolerance (one patient), intraoperative seizures (one patient), and significant emesis (one patient).

## Discussion

The evolution of AC techniques has led to their increasing adoption worldwide, supported by growing evidence of improved outcomes [19]. Our study provides additional evidence of pain relief and reduced opioid requirements in patients undergoing awake craniotomy.

While our study showed no significant difference in length of stay, previous research has demonstrated the potential economic benefits of AC, reduced intensive care unit requirements, lower overall hospital costs, decreased rehabilitation needs, and earlier return to normal activities [11,20]. In our study, the awake craniotomy group had two patients as outliers, which resulted in no statistically significant difference between these two groups. Having a larger sample size might perhaps result in a better statistical analysis.

Our finding of significantly reduced opioid requirements in the AC group aligns with the previous studies, and they further lead to a reduced risk of opioid-related complications, better pain management strategies, potential for enhanced recovery protocols, and alignment with current opioid-sparing initiatives [12,21]. Additionally, as elaborated earlier, our protocol for awake craniotomies involved the use of scalp block in all patients, and that can contribute to the decreased analgesia requirements [22].

The awake craniotomy technique does have its unique challenges and risk of complications. Due to the low number of patients in study groups, we did not perform a statistical comparison of complications between the two groups; intraoperative and postoperative complications were noted in both groups. Some intraoperative complications unique to the awake group were loss of vascular access (intravenous and arterial), airway obstruction requiring supraglottic device insertion, inability to tolerate the awake procedure, intraoperative seizures, and significant intraoperative emesis. Understanding and preparing for AC-specific complications is crucial [23]. These are mainly airway management challenges, hemodynamic fluctuations, seizure management, and emergency conversion protocols.

The operative time was also found to be longer in the awake craniotomy group. This difference may be attributed to the additional time required for awake mapping, neurological assessment by the surgeon, more meticulous resection near eloquent areas, and multiple sedation phase transitions. Further studies with larger sample sizes are needed to explore this difference.

Limitations of our study include its single-center nature and being observational. Additionally, involving a larger pool of neurosurgeons may improve the wider applications of study results. We did not look for the tumor size, which may contribute as a confounding factor. Also, the difference in ASA status between the two groups can affect the outcome.

## Conclusions

By allowing a decrease in opioid use, awake craniotomies have the potential to be considered as an alternative to traditional craniotomy while allowing surgeons to achieve the desired intraprocedural neurological assessment.

## Appendices

Anesthesia protocol: awake craniotomy/brain mapping

Preoperative Assessment

Patient selection (major medical issues (OSA, chronic obstructive pulmonary disease (COPD), cardiac disease, and severe arthritis)): Optimization is very important.

Intraoperative Management

1. Standard ASA monitors, two IVs, and arterial line (lines are placed on the same side of surgery).

2. Propofol bolus (20-100 mg) titrated to place a second IV and arterial line placement with 1% lidocaine local infiltration, and fentanyl dose (25-100 mcg) to make the patient comfortable.

3. Scalp block during this time. Anesthesia team/surgeon (scalp block by pain team solution 20 cc 0.5% ropivacaine, 9 cc 2% lidocaine with epinephrine, and 1 cc sodium bicarbonate), total of 12 sites (six on each side of the scalp), and Foley catheter.

Intraoperative Medications

1. Propofol infusion started at the beginning (25-150 mcg/kg/minute), remifentanil (0.05-0.5 mcg/kg/minute) with or without dexmedetomidine infusion (0.2-1.5 mcg/kg/hour) titrated to patient sedation.

2. The surgeon wishes for the patient to be deeper during the initial incision and craniotomy. The surgeon will notify once it is time to start waking up the patient.

3. Clevidipine or nicardipine infusions are available and ready to go to control intraoperative blood pressure.

4. Antiemetics: dexamethasone and ondansetron.

5. Keppra (1 g) and a bag of mannitol in the room. Keep 100-200 mg propofol in a 20 CC syringe to treat any seizures and 2 mg midazolam.

6. Cold saline on the surgical field to treat seizures.

Nasal airways, oral airways, LMAs, endotracheal tube (ETT), and laryngoscope/glidescope immediately available in emergency airway management.

Intraoperative Wake-Up

1. Call your attending before waking up the patient, as the patient may emerge agitated and you may need help. 

2. If used, remove the tape from the eyes/mustache/face in time. 

3. Stop all infusions 8-10 minutes before wake-up time. During the awake phase, communicate with the patient when the patient is awake and make sure the patient is comfortable.

Closure: Titrate sedation by infusions carefully, as some patients need less than at the beginning.

At the end of the procedure, the patient will go to the neurosciences intensive care unit (NSICU)/post-anesthesia care unit (PACU). 

*Postoperative* 

1. Systolic blood pressure (SBP) < 140 mmHg: use clevidipine or nicardipine infusion along with or without labetalol.
